# Cerebral Amyloid Angiopathy: Clinical Presentation, Sequelae and Neuroimaging Features—An Update

**DOI:** 10.3390/biomedicines13030603

**Published:** 2025-03-01

**Authors:** Stefan Weidauer, Elke Hattingen

**Affiliations:** Institute of Neuroradiology, Goethe University, Schleusenweg 2-16, 60528 Frankfurt am Main, Germany; hattingen@med.uni-frankfurt.de

**Keywords:** cerebral amyloid angiopathy, sequelae, intracerebral hemorrhage, magnetic resonance imaging, Boston criteria, amyloid-related imaging abnormalities, inflammation, antibody treatment

## Abstract

The prevalence of cerebral amyloid angiopathy (CAA) has been shown to increase with age, with rates reported to be around 50–60% in individuals over 80 years old who have cognitive impairment. The disease often presents as spontaneous lobar intracerebral hemorrhage (ICH), which carries a high risk of recurrence, along with transient focal neurologic episodes (TFNE) and progressive cognitive decline, potentially leading to Alzheimer’s disease (AD). In addition to ICH, neuroradiologic findings of CAA include cortical and subcortical microbleeds (MB), cortical subarachnoid hemorrhage (cSAH) and cortical superficial siderosis (cSS). Non-hemorrhagic pathologies include dilated perivascular spaces in the centrum semiovale and multiple hyperintense lesions on T2-weighted magnetic resonance imaging (MRI). A definitive diagnosis of CAA still requires histological confirmation. The Boston criteria allow for the diagnosis of a probable or possible CAA by considering specific neurological and MRI findings. The recent version, 2.0, which includes additional non-hemorrhagic MRI findings, increases sensitivity while maintaining the same specificity. The characteristic MRI findings of autoantibody-related CAA-related inflammation (CAA-ri) are similar to the so-called “amyloid related imaging abnormalities” (ARIA) observed with amyloid antibody therapies, presenting in two variants: (a) vasogenic edema and leptomeningeal effusions (ARIA-E) and (b) hemorrhagic lesions (ARIA-H). Clinical and MRI findings enable the diagnosis of a probable or possible CAA-ri, with biopsy remaining the gold standard for confirmation. In contrast to spontaneous CAA-ri, only about 20% of patients treated with monoclonal antibodies who show proven ARIA on MRI also experience clinical symptoms, including headache, confusion, other psychopathological abnormalities, visual disturbances, nausea and vomiting. Recent findings indicate that treatment should be continued in cases of mild ARIA, with ongoing MRI and clinical monitoring. This review offers a concise update on CAA and its associated consequences.

## 1. Introduction

Cerebral amyloid angiopathy (CAA) is a genetically and biochemically heterogeneous group of diseases affecting the cerebral arterial vasculature. Pathophysiologically, it is primarily characterized by impaired perivascular drainage of amyloid-beta (Aß) from the interstitial fluid (ISF) [1,2,3,4,5,6,7]. The disruption of intramural periarterial drainage (IPAD) plays a crucial role in the insufficient transport of Aß peptides via the glymphatic system into the subarachnoid space overlying the brain’s surface [3,5,8]. It is hypothesized that the glymphatic system encompasses the network of perivascular channels and their adjoining vascular and parenchymal tissue components to support the clearance of the brain’s waste into the ISF [9,10,11]. In addition to the IPAD facilitated by physiological vasomotion and cardiac pulsatility, this model includes the waste transport from the ISF towards the periarterial channels occurring along a concentration gradient, i.e., diffusion [9,10,11,12,13,14,15]. Consequently, Aß deposits accumulate in the small arterial vessel walls of the cortex and the leptomeninges, with a particular preference for parietooccipital regions [3,5,8,16]. Although histopathological investigations have also exhibited the venular accumulation of amyloid fragments, the significance of the potentially impaired venous network in CAA remains unclear [17].

The formation of Aß is catalyzed by the action of β- and γ-secretases on the amyloid precursor protein (APP) [1,3,18]. Aβ-42, which has lower water solubility compared to Aβ-40, tends to accumulate in fibrillar form within the brain parenchyma, while Aß-40 is preferentially deposited in the basement membranes of vessel walls [3,7,11,18,19]. This accumulation has been shown to reduce the mobility of smooth muscle cells, which impairs arterial pulsation and, consequently, reduces IPAD [4,5,8]. This self-perpetuating cycle leads to further Aß deposits and the expansion of the perivascular spaces (PVS) [4,5,8,16,20,21,22,23]. However, the precise mechanism behind Aß washout remains incompletely understood [18]. In contrast to CAA type 1, which is characterized by capillary deposits, CAA type 2 is not associated with such deposits [3,19,24]. Expression of apolipoprotein (APO)-Eε4 has been identified as a risk factor for type 1, whereas APO-Eε2 is linked to type 2 [3,18,24,25]. Carriers of the ε4 allele have a higher probability of developing CAA and tend to suffer more often from a severe course of the disease [18]. However, the precise mechanisms by which specific APO-E alleles manipulate the development and worsening of CAA and Alzheimer’s disease (AD) have not yet been conclusively clarified [26,27].

Other genetic risk factors facilitating CAA are presenilin 1 (PSEN1), transforming growth factor-ß1 (TGF-ß1), neprilysin, α1-antichymotrypsin, low-density lipoprotein receptor-related protein (LRP) and angiotensin-converting enzyme (ACE) [18,26,27]. Abnormalities of PSEN1 may contribute to the accumulation of Aß in the vessel walls because this gene is involved in the processing of APP and the production of Aß [18,26]. TGF-ß1 is embedded in the regulation of cell growth, tissue repair and inflammation. Genetic variations of this gene may cause alterations in its signaling pathway, resulting in the accumulation of Aß and vessel disruption [27]. Neprilysin is an enzyme which is responsible for the degradation of Aß. The acute-phase protein α1-antichymotrypsin is involved in inflammation and the regulation of proteases.

Regarding non-genetic risk factors, hypertension is a significant trigger for CAA-associated clinical features [11,18]. Persistent high blood pressure promotes weakness and partial damage of cerebral vessel walls, making them more sensitive to the degrading effects of Aß accumulation. Fibrinoid necrosis is an important degenerative sequela of hypertension in CAA and is strongly associated with the development of intracerebral hemorrhage (ICH) [18]. Table 1 provides a concise summary of the risk factors associated with the development of CAA [18,28,29,30].

Autopsy studies have shown a CAA prevalence of 5–9% in individuals aged 60–69 years, which increases progressively to 43–58% in those over 90 years of age [31,32]. While the prevalence of CAA in cognitively normal individuals aged over 80 years is 20–40%, it rises to 50–60% in those with cognitive impairment within this age group. In 90% of patients diagnosed with AD, histopathological findings also reveal CAA [31,32]. Greenberg and co-authors [3] summarized this phenomenon as ‘two converging courses–one peptide’. Neurologically, CAA often manifests as spontaneous lobar ICH [1,3], with a high risk of recurrence, transient focal neurologic episodes (TFNE) [33,34,35,36] and progressive cognitive decline ranging from mild cognitive impairment up to AD [1,25].

The definitive diagnosis of CAA still requires histologic evidence. The Boston criteria first described in 1995 enable the diagnosis of a probable or possible CAA by considering defined neurological and magnetic resonance imaging (MRI) findings [37]. The current version 2.0 with additional non-hemorrhagic MRI findings increases the sensitivity with unchanged specificity (Table 2) [38].

## 2. Clinical Presentations of CAA

**Figure 1:**
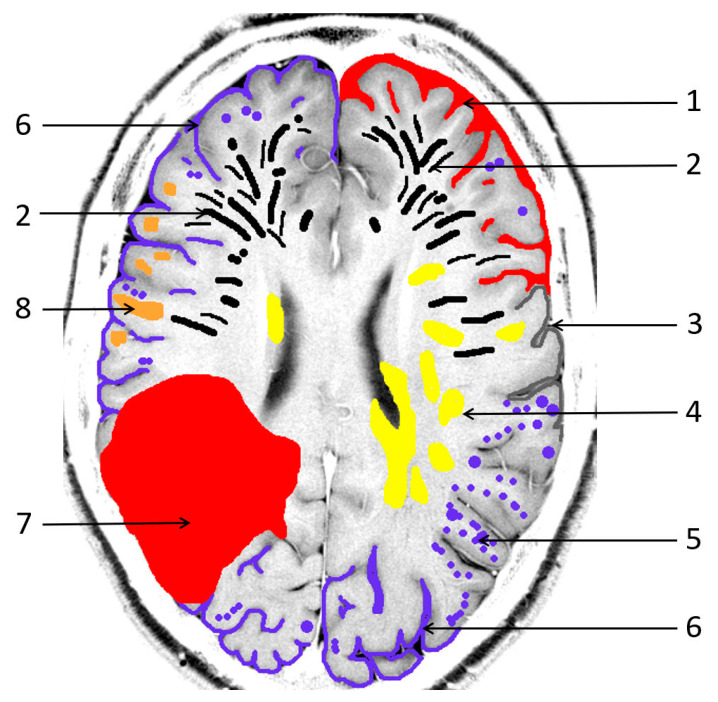
Pathological sequelae due to cerebral amyloid angiopathy (CAA). 1: cortical subarachnoid hemorrhage (cSAH); 2: enlarged/severe centrum semiovale perivascular spaces (CSO PVS); 3: focal cortical thinning; 4: white matter hyperintensities in a multispot pattern (WMH MS); 5: cortical microbleeds (MB); 6: cortical superficial siderosis (cSS); 7: lobar intracerebral hemorrhage (ICH); 8: cortical/subcortical lacunar infarct.

**Figure 2:**
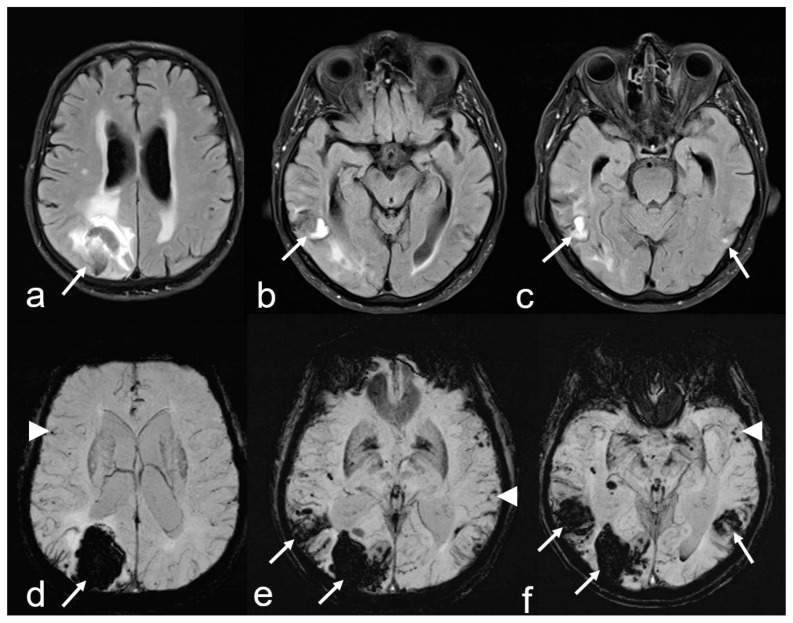
Recurrent intracerebral hemorrhages (ICH) within 2 years in a 74-year-old man with a history of progressive cognitive impairment. The patient was on antihypertensive medication and a statin but had neither antithrombotic drugs nor anticoagulant therapy. The final diagnosis was new lobar ICB due to probable CAA in accordance with the 2.0 version of the Boston criteria [38]. Axial FLAIR (fluid attenuated inversion recovery) images (**a**–**c**) showing three ICHs at different time points parieto-occipital right ((**a**,**d**): arrow), temporal right ((**b**–**e**): arrow) and temporal left ((**c**,**f**): arrow); (**d**–**f**): susceptibility-weighted imaging (SWI; arrow) disclosing additional multiple microbleeds (MB) with temporal accentuation (**d**–**f**: arrowhead); MRI 1.5 T Siemens AREA.

The most common acute neurological manifestation of CAA is lobar ICH, which carries a high risk of recurrence [1,2,3] (Figure 1 and Figure 2). Particularly in elderly patients over 75 years of age, spontaneous ICH is associated with a high mortality rate and higher blood glucose levels, and the neutrophil to lymphocyte ratio indicates an increased risk of short-term death [39,40,41,42]. Additionally, neurologically recurrent, stereotypical unilateral sensory, motor or speech deficits that extend to different parts of the body are noteworthy. These episodes, referred to as TFNE or ‘amyloid spells’ [1,33,34,35,36], should not be confused with focal epileptic seizures (e.g., sensory or motor Jackson seizure) or transient ischemic attacks (TIA) [36]. The etiology of these episodes is often linked to cortical subarachnoid hemorrhages (cSAH), which typically occur near the vertex above the convexity [43,44,45,46,47], or cortical superficial siderosis (cSS) [33]. Cortical spreading depolarization has been identified as a pivotal pathophysiological process [33]. Another significant neurological manifestation is a slowly progressive cognitive progressive cognitive decline, which can eventually lead to AD [3,25,31,33].

## 3. Neuroimaging Features

The imaging characteristics of CAA are summarized in Figure 1.

**Figure 3:**
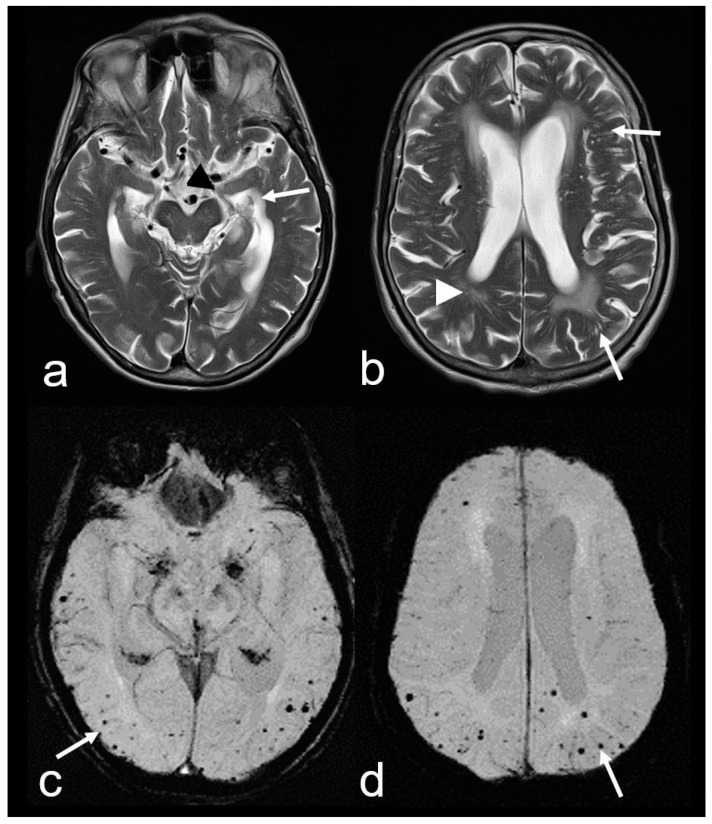
Cerebral amyloid angiopathy (CAA) and Alzheimer’s disease (AD) in an 82-year-old woman with progressive cognitive decline and short-term memory disturbance. Axial T2-weighted images (WI) (**a**,**b**) showing distinct temporal mesial atrophy ((**a**), arrowhead), enlarged temporal horns ((**a**), arrow), vascular leukoencephalopathy ((**b**), arrowhead) and enlarged perivascular spaces (PVS; (**b**), arrow). Susceptibility-weighted imaging (SWI) ax. (**c**,**d**) disclosing multiple cortical and subcortical microbleeds (MB) (arrow), especially temporal and parietal; MRI 1.5 T Siemens AREA.

**Figure 4:**
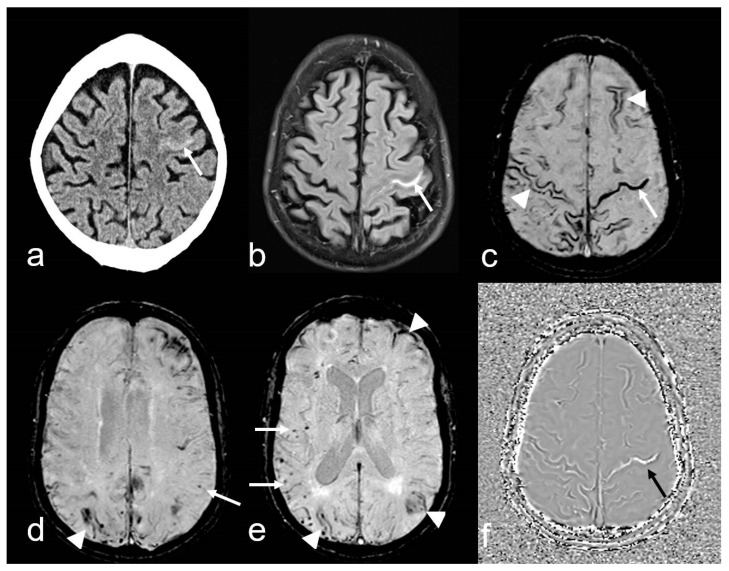
A 74-year-old man with progressive cognitive impairment suffering from temporary sensory–motoric deficits right. Cortical subarachnoid hemorrhage (cSAH) in the central sulcus ((**a**), CT: arrow; Siemens Somatom Emotion). MRI with sulcal hyperintense signal changes on fluid attenuated inversion recovery (FLAIR) images ((**b**), arrow), sulcal signal loss on susceptibility-weighted imaging (SWI, (**c**): arrow), additional multifocal cortical superficial siderosis (cSS) bilateral ((**c**–**e**), arrowhead); note the characteristic bilinear track-line appearance of cSS in the chronic stage ((**c**), arrowhead); (**d**,**e**): multiple cortical/subcortical microbleeds (MB, arrow); (**f**): SWI-phase image demonstrating paramagnetic effects in the central sulcus due to blood degeneration products (arrow); MRI 1.5 T Siemens AREA.

1. *Intracerebral hemorrhages (ICH)* are the most common manifestation of CAA, with lobar ICH being the most prevalent [1,3,18]. These are associated with a high risk of recurrence, particularly in the temporoparietal and occipital regions (Figure 2) [1,18]. Histopathological analyses in post-mortem studies have demonstrated subcortical white matter changes in a multispot pattern and in cortical infarcts due to CAA complete replacement of the vascular smooth cell layer by Aß deposits (Vonsattel grade 2) [48,49,50]. Moreover, individual vessels associated with ICH specimens showed complete vascular remodeling in the form of Vonsattel grade 3–4 [48,49,50,51,52,53]. While APOE-E4 has been identified as risk factor for recurrent CAA-associated bleeding, APOE-E2 is the primary risk factor for AD and CAA, often leading to a more severe clinical course [3,18,24,25]. Neuroimaging biomarkers associated with the highest risk of further ICH in patients suffering from previous CAA-related ICH are (a) a higher number of cerebral microbleeds (cMB), (b) disseminated or multifocal cSS and (c) the development of new cSS on follow-up MRI. Therefore, ICH prediction also has an impact on the risk-versus-benefit calculation of antithrombotic treatment in patients with indications such as atrial fibrillation [51,52]. Restarting of platelet aggregation inhibitors, i.e., acetylsalicylic acid, seems to be a reasonable safe option following ICH. However, a clear and ambiguous individual indication should be a requirement for anticoagulant therapy in these patients. To date, the extent to which patients with CAA-associated ICH will benefit from anticoagulation in nonvalvular atrial fibrillation regarding the prevention of stroke (ischemia versus hemorrhage) has not been conclusively clarified [40,51].

2. *Cortical and subcortical microbleeds (MB)*, which have been identified as a risk factor for lobar ICH and ischemia, have also been associated with cognitive impairment [38,53,54,55]. In contrast to hypertensive microangiopathy caused by lipohyalinosis, which typically affects the basal ganglia, thalamus, pons and cerebellum [55,56,57,58,59,60], MB are generally absent in these regions in CAA [38,54,61]. Due to the neurodegenerative and cerebrovascular processes associated with Aß deposits, MBs are preferentially localized in the parieto-occipital regions in both CAA and AD (Figure 1, Figure 2 and Figure 3) [3,19,53,54,61].

3. *Cortical SAH* (cSAH) is frequently localized in the frontodorsal and central regions over the convexity near the vertex [43,44,45,46] (Figure 4). CAA is, by far, the most frequent cause of cSAH, especially in older individuals, and may occur repeatedly at different locations (see Figure 4) [46,62,63]. However, the etiology of cSAH also includes peripheral aneurysms, arteriovenous malformation (AVM), dural fistula, reversible cerebral vasoconstriction syndrome (RCVS) and tumors or preceding high-grade arterial stenosis, vasculitis and venous outflow obstruction [45,46,62,64]. Therefore, comprehensive clinical and neuroradiological assessments are essential. Clinical signs are often TFNE (‘amyloid spells’) [1,33,34,35,36]. The patients typically experience multiple episodes of contralateral sensory disturbances, each lasting approximately 15 min, often occurring within a 24 h period [33,34]. From a neurological perspective, differential diagnosis in an emergency setting can be challenging, as it may be difficult distinguish these episodes from TIAs or focal epileptic seizures, such as sensory Jackson seizures [36].

4. *Cortical superficial siderosis (cSS)* refers to the presence of linear hemosiderin in the leptomeninges and the superficial layers of the cerebral cortex, most commonly as a sequela of a previous acute cSAH [62,65,66,67,68,69,70]. It is important to differentiate between localized cSS, which affects 1–3 sulci, and disseminated cSS, which affects at least 4 sulci [70]. In the acute stage, T2*-weighted imaging (WI) and susceptibility-weighted imaging (SWI) typically show homogeneous signal loss, while in the chronic stage, a bilinear, “track-line” appearance is characteristic (Figure 4) [63,65,66].

In contrast, infratentorial SS is often due to chronic intermittent or continuous slight bleeding into the subarachnoid space [62,65,66,71], and the most common etiology is spinal dural abnormality, e.g., dural tears [72,73]. Infratentorial SS involve the cerebellum, brainstem and cranial nerves, especially the vestibulocochlear nerve, and the spinal cord [71,74,75].

**Figure 5:**
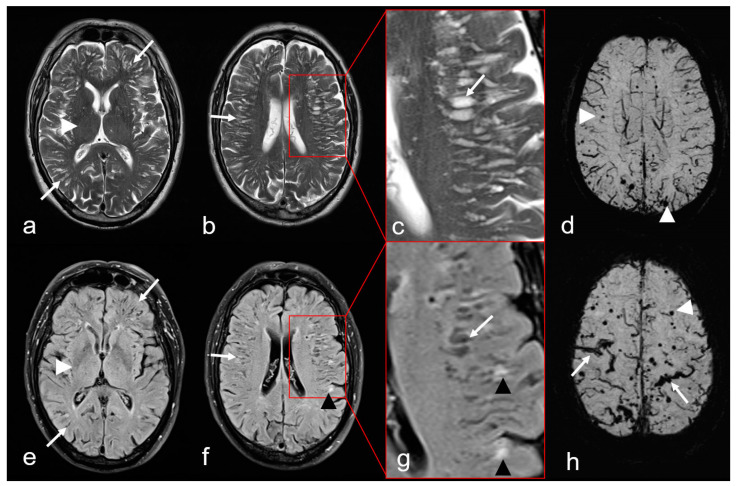
Non-hemorrhagic and hemorrhagic MRI features in cerebral amyloid angiopathy (CAA). Severe enlarged perivascular spaces (PVS) supratentorial ((**a**–**c**): T2-weighted images (WI), arrow); (**e**–**g**): Fluid attenuated inversion recovery (FLAIR) images (arrow) sparing the basal ganglia ((**a**,**e**): white arrowhead), characteristic for a centrum semiovale (CSO) PVS pattern. (**f**,**g**): Multiple partially conflating white matter hyperintensities in a multispot pattern (black arrowhead, WMH-MS). (**d**,**h**): Susceptibility-weighted images (SWI) exhibit additional hemorrhagic lesions, i.e., multiple cortical/subcortical microbleeds (MB, white arrowhead) and multifocal cortical superficial siderosis (cSS; (**h**), arrow); MRI 1.5 T Intera, Philips Healthcare.

5. *Non-hemorrhagic supratentorial white matter changes*:

(a) The presence of enlarged PVS in the centrum semiovale (CSO), sparing the basal ganglia and thalamus, i.e., CSO PVS (Figure 5) [38,76].
(b) Multiple hyperintense lesions in the white matter/CSO (WMH MS: white matter hyperintensities multispot) as depicted on T2-weighted sequences (Figure 5) [38,76,77,78,79].

Furthermore, a focal decrease in cortex thickness and subcortical or cortical small infarcts are also detected as a consequence of CAA [80,81,82]. However, these findings are not yet included in the Boston criteria version 2.0 for the clinical diagnosis of CAA (see Figure 2 and Table 2) [38]. The differential diagnosis of band-shaped leptomeningeal/sulcal and circumscribed cortical/subcortical signal loss in hemosensitive MRI sequences (CAA mimics) are summarized in Table 3 [62,83]. However, a detailed description of CAA mimics is beyond the scope of this review, and readers are referred to the relevant literature.

## 4. Advanced Imaging Techniques

Dynamic contrast-enhanced MRI (DCE-MRI) after intrathecal contrast agent (CA) administration and subsequent T1-weighted MRI scans at intervals of 3–6 h over a period of 2 days is the current gold standard for measuring glymphatic transport [11,84,85]. However, this technique is invasive, relies on off-label use of the gadolinium-based contrast agents and is not practicable for human routine use. Also, heavily T2-weighted 3D fluid-attenuated inversion recovery (FLAIR) images with delayed acquisition 4 h after intravenous (iv.) CA administration may be used [84,85]. Diffusion tensor imaging along the perivascular spaces (DTI-ALPS) as a method not dependent on CA application may show a decreased DTI-ALPC index due to the enlargement of PVS with consecutive altered perpendicular orientation of major white matter tracts in a region next to the ventricles. However, it is not clear whether this method accurately measures glymphatic activity and therefore the results should be interpreted with caution [11,84].

## 5. Boston Criteria

The definitive diagnosis of CAA can only be made post-mortem and requires histological evidence of a univocal CAA with concomitant vasculopathy [1,2,3,38], the exclusion of other diagnostic lesions and previous clinical presentation with either spontaneous ICB, cSAH and neurologically cognitive decline or even dementia or TFNEs (Table 2) [33,34,35,36,38].

The Boston criteria were first formulated in 1995 [2], with version 1.0 established in 2001 [37] to enhance structured diagnostic classification. In addition to neurological findings, these criteria included specific MRI findings, such as the detection of ≥2 hemorrhagic lesions, including a lobar spontaneous ICH and cortical or subcortical MBs (Figure 1, Table 2) [37]. In 2010, Linn and co-authors [64] demonstrated that 60.5% of patients with a CAA exhibited a cSS, a feature not observed in the control group. The subsequent modified version 1.5 of the Boston criteria, with the incorporation of focal (≤3 sulci affected) or disseminated (≥ 4 sulci involved), enhanced the sensitivity from almost 90% to 94.7%, while maintaining unaltered specificity [37,86].

The current version 2.0 [38] also incorporates also non-hemorrhagic MRI findings, such as multiple T2-weighted supratentorial WMH MS and markedly enlarged PVS in the CSO, excluding the basal ganglia [38,76,77,78]. These revised criteria for probable CAA were found to be significantly more closely associated with histologic evidence of CAA compared to version 1.5 [38,87,88,89]. A noteworthy addition is that cSS, when present as the sole hemorrhagic feature in combination with a non-hemorrhagic criterion and a corresponding clinical presentation, now allows the diagnosis of probable CAA [38]. Furthermore, the minimum age for diagnosis was lowered to 50 years (Table 2) [36,38,64].

Even though the Boston criteria version 2.0 and the Edinburgh criteria facilitate the diagnosis of possible or probable CAA in daily clinical practice [38,87,88,89], the gold standard remains biopsy or even autoptic confirmation [38]. It is also important to note that incidental neuroradiologically typical CAA findings have been detected in approximately 16% of clinically asymptomatic older individuals [8,31,32,54,90,91]. In contrast, neurological symptoms, particularly those classified as TFNE, have been shown to manifest several years before the diagnosis of CAA [36]. This highlights the need for prompt evaluation using hemosensitive MRI sequences, such as T2*-weighted sequences and high-resolution SWI [36,91,92,93,94].

## 6. CAA-Related Inflammation (CAA-ri)

**Figure 6:**
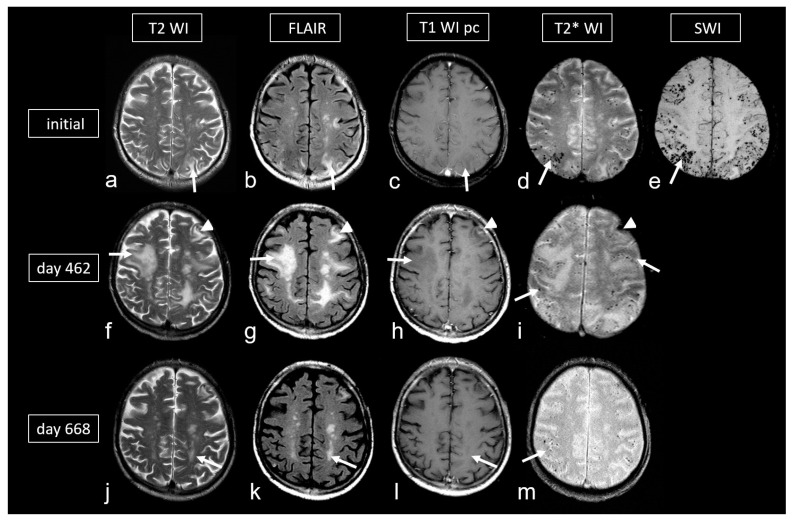
Cerebral amyloid angiopathy-related inflammation (CAA- ri) in a 72-year-old man suffering from subacute deterioration of consciousness and dizziness. (**a**–**e**): Initial MRI (upper row; MRI 3.0 T Siemens Magnetom) showing several hyperintense lesions preferentially in the subcortical occipital region ((**a**,**b**): arrow) without contrast enhancement on post-contrast T1-weighted images (T1 WI pc; (**c**), arrow), focal-accentuated microbleeds (MB) on T2* WI ((**d**), arrow) and susceptibility-weighted imaging (SWI; (**e**), arrow); note the higher sensitivity for MB on SWI (**e**) compared to T2* WI (**d**). (**f**–**i**): MRI (1.5 T Intera, Philips Healthcare) at readmission due to subacute severe psychosyndrome after tapered corticosteroid therapy. Multiple occasionally space-occupying hyperintense white matter lesions ((**f**,**g**): arrow) without contrast enhancement ((**h**), arrow) and progressive bilateral MBs ((**i**), arrow). Note the additional subacute small left frontal intracerebral hemorrhage (ICH; arrowhead). (**j**–**m**): Follow-up MRI (1.5 T Intera, Philips Healthcare) after several bouts of intravenous high-dosage methylprednisolone showing distinct regression of white matter lesions ((**j**,**k**): arrow) without contrast enhancement ((**l**), arrow), no significant new hemorrhagic lesions (**m**).

CAA-related inflammation (CAA-ri) is a subtype of CAA characterized by the presence of autoantibodies against Aß in the arterial vessel walls [95,96,97,98,99,100]. The inflammation, which affects both the vascular and perivascular regions, leads to vasogenic edema and leptomeningeal effusions, resulting in hyperintense signal changes on T2-weighted sequences [96,100]. These MRI features are identical to the imaging changes observed during amyloid antibody therapy [97,101,102], in which these are known as “amyloid related imaging abnormalities” (ARIA) (Figure 6) [101,102,103,104,105]. ARIA-E (edema, effusion), characterized by circumscribed peripheral edema often involving the cortex [101,106,107], is frequently accompanied by MBs, cSAH or cSS (ARIA-H, hemorrhagic) resulting in overall picture that aligns with CAA-ri, making both entities morphologically indistinguishable [101,103,104,105].

**Figure 7:**
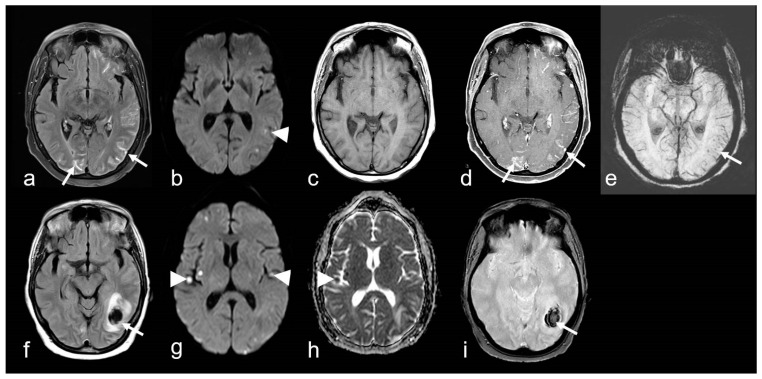
A 68-year-old woman suffering from progressive dizziness and visual blurring for several weeks (upper row) and acute deterioration (lower row) due to cerebral amyloid angiopathy-related inflammation (CAA-ri) with associated vasculitis (amyloid-beta-related angiitis, ABRA). (**a**–**e**): Multifocal hyperintense sulcal effusions ((**a**), arrow; fluid-attenuated inversion recovery (FLAIR)), focal small lesions with restricted diffusion temporo-parietal left ((**b**), arrowhead; diffusion-weighted imaging (DWI, b = 1000 s/mm^2^)), distinct multifocal leptomeningeal enhancement ((**d**), arrow; (**c**,**d**): T1 WI before (**c**) and after (**d**) contrast agent application); (**e**): multiple microbleeds (arrow, susceptibility-weighted imaging (SWI)). (**f**–**i**): Subacute lobar intracerebral hemorrhage (ICH; (**f**), arrow); (**g**,**h**): new cortical / subcortical infarcts ((**g**,**h**): arrowhead; DWI, b = 1000 s/mm^2^, apparent diffusion coefficient (ADC) map); (**g**): T2* WI demonstrating inhomogeneous signal loss (arrow); MRI 1.5 T Intera, Philips Healthcare.

**Figure 8:**
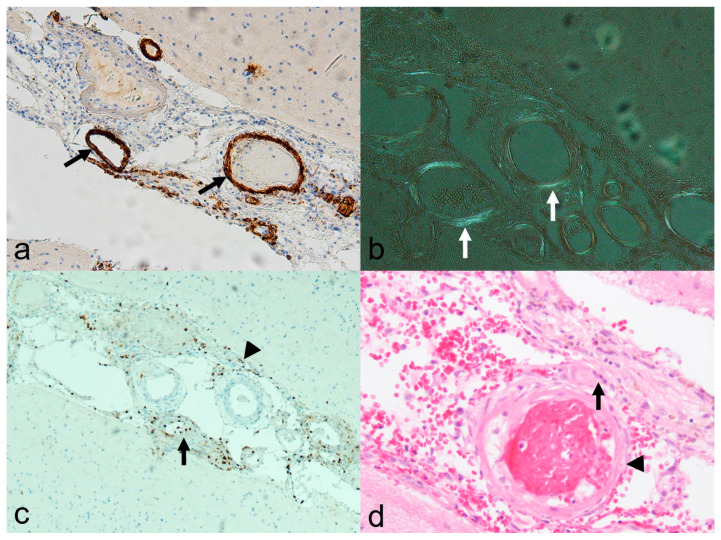
Histological specimen in cerebral amyloid angiopathy-related inflammation (CAA-ri) and associated vasculitis (amyloid-beta-related vasculitis, ABRA; biopsy of the pat. showed in Figure 7). (**a**): Beta A4 amyloid staining (10×) showing distinct immune histochemical evidence of beta amyloid (brown colored, arrow) in the vessel wall; (**b**): typical “apple green” color due to birefringence in polarized light (arrow; Congo-red staining, 10×); (**c**): leukocyte common antigen (LCA) staining (10×) disclosing lymphocytic infiltration in the arterial walls (arrow) and the leptomeninx (arrowhead); (**d**): hematoxylin-eosin staining (20×), also showing multinucleated giant cell (arrow) adjacent to the vessel wall (arrowhead). Courtesy L. Schweizer, Edinger–Institute, Neuropathology, Goethe University, Frankfurt.

Histological examination revealed amyloid deposits, accompanied by lymphocytic infiltrations, either perivascular or within the vessel wall itself [97,99,105]. The latter feature has been categorized as Aß-associated vasculitis (ABRA) (Figure 7 and Figure 8) [108,109]. However, in more recent studies, no distinction has been made between these two variants [89]. Nevertheless, primary CNS angiitis (PCNSA) must be differentiated from ABRA due to the presence of inflammatory vessel wall infiltrates without amyloid deposits on histological examination [96,109,110,111,112,113].

Neurologically, CAA-ri typically presents with rapidly progressive psychosyndromes, along with cognitive impairment, changes in consciousness, headaches, epileptic seizures and, depending on the affected area, focal neurological deficits [96,97,98]. Similar to the Boston criteria, clinical and imaging parameters have been established to diagnose possible or probable CAA-ri (Table 4) [96].

In probable CAA-ri, MRI shows one or more asymmetric T2-hyperintense lesions in the subcortical or deep white matter, extending directly to the subcortical region. These lesions are not attributable to a previous ICH [96]. Furthermore, the presence of at least one hemorrhagic lesion (MB, ICH or cSS) is a mandatory inclusion criterion (Figure 6 and Figure 7) [96]. Neoplastic, infectious or other potential etiologies must be excluded. In contrast to the Boston criteria version 2.0 [38], the minimum age for diagnosis is set at 40 years [96]. Knowledge of these findings is crucial, as a biopsy is required for definitive confirmation of the diagnosis. In spontaneous CAA-ri, initial iv. Cortisone pulse therapy with 1000 mg/day for at least 3–5 days with subsequent slow tapering over several months leads to a good or even complete regression of the neurological deficits in 84% of cases (Figure 6) [114,115,116,117]. Focal atrophy can also occur if the anti-inflammatory therapy does not respond [98,114].

## 7. Monoclonal Antibody Therapies Against Amyloid Deposits in Alzheimer’s Disease (AD)

The pathogenesis of AD has not been clarified, and the role of the amyloid deposits in relation to the clinical symptoms remains unclear [3,4,5]. However, several monoclonal antibodies (AB) aimed at clearing Aß deposited from the brain parenchyma or reducing Aß peptides by inhibiting APP cleavage have been investigated in phase III studies [105,118,119,120,121,122,123]. Following the additional evaluation of the studies involving the monoclonal AB aducanumab (EMERGE, EN-GAGE, Biogen; Cambridge, MA 02142, USA), it was approved by the Food and Drug Administration (FDA) under the accelerated approval pathway in the United States as a treatment option for patients with mild cognitive impairment or mild AD, subject to follow-up evaluations [105,122]. However, further discussion of additional studies is beyond the scope of this review, and readers are referred to the relevant literature.

**Figure 9:**
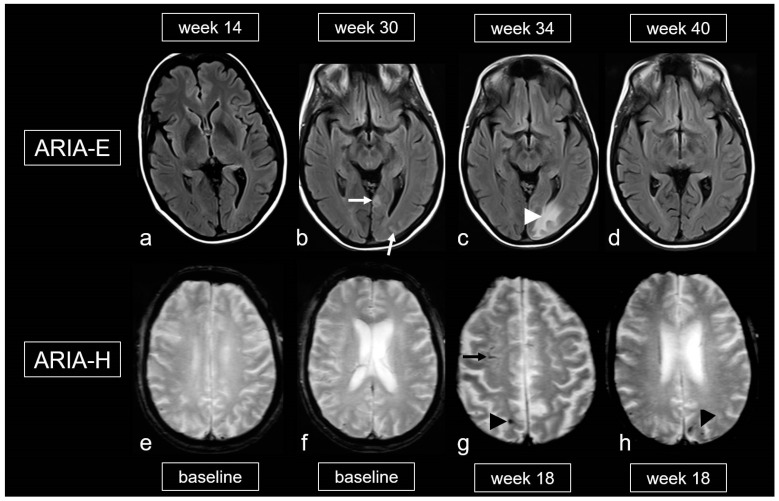
Amyloid-related imaging abnormalities (ARIA). (**a**–**d**): Fluid-attenuated inversion recovery (FLAIR) images showing ARIA-E (edema, effusion) in a patient treated with aducanumab, week 14 (**a**), 30 (**b**), 34 (**c**) and 40 (**d**) after treatment initiation; sulcal effusions ((**b**), arrow) and edema in the occipital lobe ((**c**), arrowhead), completely decreased at week 40 (**d**). (**e**–**h**): T2*-weighted images (WI) demonstrating ARIA-H (hemorrhagic) in a 68-year-old woman treated with aducanumab, baseline (**e**,**f**) and week 18. (**g**,**h**): Cortical superficial siderosis (cSS; arrow) and microbleeds (MB; arrowhead); MRI 1.5T.

Biochemically, there is an increased conversion of the less water-soluble Aß-42, located in the parenchyma, into the more soluble Aß-40. This leads to a significantly higher concentration of Aß-40 in the perivascular drainage system, resulting in overload and potential damage to smooth muscle cells, among other effects [3,4,5,101,119,122]. This can cause extravasation of protein-rich fluid, leading to the development of edema and leptomeningeal effusions (ARIA-E). The extravasation of erythrocytes may result in ARIA-H (see Figure 9) [101,105].

Finally, the literature also mentions an iatrogenic variant of CAA-ri related to the frequent and dose-dependent occurrence of ARIA, which affects up to 41.3% [101,105,124,125]. The gradations of ARIA-E and ARIA-H are outlined in Table 5 and Table 6 [105,126]. It is important to note that, unlike spontaneous CAA-ri, only about 20% of patients with proven ARIA on MRI exhibit clinical symptoms, such as headache, confusion and other psychopathological abnormalities, visual disturbances, nausea and vomiting [101,105,124,125,126]. These symptoms typically arise within the first 3 months of treatment and are usually reversible.

Understanding these processes, the neuroradiological constellation of imaging findings and the precise characterization of the ARIA are crucial [105,126]. This is important because adjustments to MRI examination intervals may be required, and recent findings suggest that treatment should be continued in cases of mild ARIA [126].

In contrast to the promising therapeutic approaches for the treatment of AD, anti-Aß AB immunotherapy has not yet shown any significant breakthrough with regard to CAA-associated vascular alterations, e.g., vascular remodeling [51,127]. This corresponds to both non-hemorrhagic and hemorrhagic sequelae including, especially ICH [51]. Further studies with candidate targets, including Aß production and clearance, are necessary. However, lipophilic short-interfering ribonucleic acids (RNAs) and the stimulation-induced entrainment of neurovascular oscillations might reflect promising new therapeutic approaches for manipulating central nervous systems processes in CAA [51,128,129].

## 8. Conclusions

CAA due to vascular Aß accumulation occupies a highly prominent position both with regard to the demographic changes with increasing aging of the population and the subsequent risk of lobar ICH and AD, as well as in terms of physiological assessment and analysis of the glymphatic system [3,38,40,51]. The three models of glymphatic perivascular waste clearance encompass the network of perivascular channels and their adjoining vascular and parenchymal tissue components [10,11,14]. In addition to the traditional glymphatic hypothesis, recent studies in rodents have indicated that IPAD facilitated by vasomotion particularly during sleep plays a key role in driving fluid within the glymphatic system to support the clearance of Aß from the brain into the ISF [10,11]. In addition, in the mixing model, bulk flow is present only on the surface of the brain along the large pial arteries, which provides an effective concentration gradient for waste exit by diffusion from the ISF to the arterial PVS [14].

The impairment of perivascular clearance by Aß deposition initiates a self-perpetuating cycle leading to further Aß deposits, subsequent expansion of the PVS and progressive degeneration of the vessel walls. Consecutive vascular remodeling facilitates the recurrence of lobar ICH.

However, up to now, it remains unclear whether primary glymphatic dysfunction results in consecutive vascular Aß accumulation, or whether initial Aß deposition of another alternative cause induces impaired clearance [11,14]. Therefore, further investigations, including rodent DCE-MRI and novel integrated computational models serving as a generalized framework, are necessary to link findings and mechanistic insights from the rodent brain to the wide anatomical scale of the human brain [10,52]. Further clarification of the impaired glymphatic function is also of great interest with regard to ARIA following anti-Aß immunotherapy [125,126].

In addition to known neuroradiological biomarkers, the identification of novel imaging biomarkers, especially with regard to non-invasive imaging procedures, e.g., heavily T2-weighted 3D FLAIR images with delayed acquisition after intravenous CA administration or DTI-ALPC, might be helpful in detecting early stages of the disease [84,85]. Furthermore, as the neurological overture of CAA may occur several years before clinical diagnosis, early clarification by MRI including hemosensitive sequences is recommended [36].

Although disease-modulating therapies have recently been established in AD [130], the same strategic approach of anti-Aß immunotherapy in CAA has so far failed to improve vascular function [11,51]. However, lipophilic short-interfering ribonucleic acids (RNAs) and stimulation-induced entrainment of neurovascular oscillations might reflect promising new therapeutic approaches [128,129]. Further early-phase studies are necessary to clarify the possible clinical effectiveness of these therapeutic approaches.

## 9. Key Messages

➢ CAA is characterized by amyloid deposits in small cortical and leptomeningeal vessels.
➢ The prevalence of CAA increases with age, reaching 50–60% in cognitively impaired individuals over the age of 80.
➢ A frequent manifestation is spontaneous lobar ICH, which carries a high risk of recurrence.
➢ Typical neurological signs are TFNE and progressive cognitive deficits up to AD.
➢ Characteristic neuroradiological findings are (a) hemorrhagic: lobar ICH, cortical/subcortical MB, cSAH, cSS; (b) non-hemorrhagic: enlarged CSO PVS and multispot pattern of T2-hyperintense lesions in the centrum semiovale (WMH MS)
➢ Hemosensitive MRI sequences (T2*, better: SWI) are essential.
➢ The Boston criteria version 2.0 facilitate the clinical diagnosis.
➢ CAA-ri is a subtype that presents in two variants of MRI features, which are identical to the changes observed during amyloid AB therapy: (a) ARIA–edema and leptomeningeal effusion (ARIA-E); (b) hemorrhagic lesions (ARIA-H).
➢ ARIA occur in approximately 20% of patients undergoing monoclonal AB therapy against Aß.

## Figures and Tables

**Table 1:** Risk factors for the development of cerebral amyloid angiopathy (CAA) [18].

Genetic Risk Factors		Non-Genetic Risk Factors
Polymorphisms	Apoliporotein E (epsilon2/epsilon4)	Amyloid beta protein (Aß)–immunotherapies *
	Transforming growth factor ß1	Iatrogenic CAA (neurosurgical procedures)
	Presenilin 1	Radiation therapy
	Alpha 1-antichymotrypsin	Hypertension
	Neprilysin	Brain injury
	Low-density lipoprotein receptor-related protein	
	Angiotensin converting enzyme	
Mutations of the CAA-related genes (Amyloid Precursor Protein/presenilins)		

Abbreviations: CAA: cerebral amyloid angiopathy. *: is also referred to by some authors as iatrogenic CAA (therapy-associated).

**Table 2:** Boston criteria version 2.0 for sporadic cerebral amyloid angiopathy (CAA) [38].

Level	Determination	Criteria
**Definite/proven CAA**	Full postmortem examination	Presentation with spontaneous lobar ICH, TFNEs, cSAH or cognitive impairment/dementia
		Severe CAA with vasculopathy
		Absence of other diagnostic/trendsetting lesion
**Probable CAA with supporting pathology**	Clinical data and pathological tissue (received from evacuated hematoma or cortical biopsy)	Presentation with spontaneous lobar ICH, TFNEs, cSAH or cognitive impairment/dementia
		Histological evidence/some degree of CAA in specimen
		Absence of other diagnostic/trendsetting lesion
**Probable CAA**	Clinical data and MRI	Age ≥ 50 years
		Presentation with spontaneous lobar ICH, TFNEs or cognitive impairment/dementia
		≥2 of the strictly lobar hemorrhagic lesions on MRI/T2* WI (SWI), in any combination:
		ICH, cMB, cSS/cSAH foci
		or
		1 lobar hemorrhagic lesion (ICH, cMB, cSS, cSAH)
		and
		1 defined white matter feature (severe CSO-PVS or WMH MS)
		Absence of any deep hemorrhagic lesions (ICH, cMB) on MRI/T2* WI (SWI)
		Absence of other cause of hemorrhagic lesions (#)
		Hemorrhagic lesion in the cerebellum not counted as either lobar or deep hemorrhagic lesion
**Possible CAA**	Clinical data and MRI	Age ≥ 50 years
		Presentation with spontaneous lobar ICH, TFNEs or cognitive impairment/dementia Absence of other cause of hemorrhagic lesions (#)
		1 of the strictly lobar hemorrhagic lesions on MRI/T2* WI (SWI): ICH, cMB, cSS/cSAH foci
		or
		1 defined white matter feature (severe CSO-PVS or WMH MS)
		Absence of any deep hemorrhagic lesions (ICH, cMB) on MRI/T2* WI (SWI)
		Absence of other cause of hemorrhagic lesions (#)
		Hemorrhagic lesion in the cerebellum not counted as either lobar or deep hemorrhagic lesion

Abbreviations: CAA: cerebral amyloid angiopathy; cMB: cerebral microbleeds; CSO-PVS: centrum semiovale–perivascular spaces; cSAH: cortical subarachnoid hemorrhage; cSS: cortical superficial siderosis; ICH: intracerebral hemorrhage; MRI: magnetic resonance imaging; SWI: susceptibility-weighted images; TFNE: transient focal neurologic episode; WI: weighted images; WMH MS: white matter hyperintensities multispot; #: other causes of hemorrhagic lesions: head/brain trauma, hemorrhagic transformation of an ischemic infarct, arteriovenous malformation, hemorrhagic tumor, central nervous system vasculitis, other causes of cSS or cSAH.

**Table 3:** Mimics of CAA. Differential diagnosis of linear leptomeningeal/sulcal or circumscribed cortical/subcortical signal loss on hemosensitive MRI sequences (T2*-weighted images, susceptibility-weighted images (SWI)).

Leptomeningeal/Sulcal	Cortical/Subcortical
cortical vein and/or sinus thrombosis	cavernoma (*)
laminar cortical necrosis	parasitosis * (e.g., toxoplasmosis, neurocysticercosis, other)
cortical hemorrhagic transformation of ischemic infarct	micrometastasis (e.g., melanoma, other)
SAH of different cause than CAA	“flow void“ phenomenon of small vessels
aneurysmal SAH	traumatic axonal injury (DAI)
vasculitis	ECMO
arterial proximal high-grade stenosis	microbleeds of different cause than CAA
RCVS	CADASIL
AVM	vasculitis
trauma	lipohyalinosis
tumor	post radiation
other	
Sturge–Weber syndrome *	
calcificating angiopathy *	
Cockayne syndrome *	

Abbreviations: AVM: arteriovenous malformation; CAA: cerebral amyloid angiopathy; CADASIL: cerebral autosomal dominant arteriopathy with subcortical infarcts and leukoencephalopathy; DAI: diffuse axonal injury; ECMO: extracorporeal membrane oxygenation; RCVS: reversible cerebral vasoconstriction syndrome; SAH: subarachnoid hemorrhage; *: in susceptibility-weighted imaging (SWI), the phase image is negative due to the diamagnetic effect of the calcifications (in contrast to the positive-phase image with a paramagnetic effect due to bleeding; see Figure 4f).

**Table 4:** Diagnostic criteria of CAA-related inflammation (CAA-ri) [96].

Level	Criteria
**Definite/proven CAA-ri**	Histopathological evidence of cerebral amyloid angiopathy
	and
	Perivascular/transmural and/or intramural inflammation/lymphocytic infiltrates (Aß angiitis)
**Probable CAA-ri**	Age ≥ 40 years
	Presence of ≥1 clinical feature, i.e.,:
	Headache
	Decrease/impairment of consciousnss
	Behavioral change
	Focal neurological deficits
	Seizures
	Symptoms not directly attributable to an acute intracerebral hemorrhage
	MRI: uni- or multifocal white matter hyperintensities cortical/subcortical or deep, asymmetric and extend to the juxtacortical white matter; asymmetry not caused by former intracerebral hemorrhage
	MRI: ≥1 cortical/subcortical hemorrhagic lesion, i.e.:
	Cerebral macrobleed (ICH)
	Cerebral microbleed
	Cortical superficial siderosis
	Absence of neoplastic, infectious or other causes
**Possible CAA-ri**	Age ≥ 40 years
	Presence of ≥ 1 clinical feature, i.e.,:
	Headache
	Decrease/impairment of consciousnss
	Behavioral change
	Focal neurological deficits
	Seizures
	Symptoms not directly attributable to an acute intracerebral hemorrhage
	MRI: white matter hyperintensities extending to the juxtacortical white matter
	MRI: ≥1 cortical/subcortical hemorrhagic lesion, i.e.,:
	Cerebral macrobleed (ICH)
	Cerebral microbleed
	Cortical superficial siderosis
	Absence of neoplastic, infectious or other causes

Abbreviations: Aß: amyloid beta; CAA-ri: cerebral amyloid angiopathy-related inflammation; ICH: intracerebral hemorrhage; MRI: magnetic resonance imaging.

**Table 5:** Graduation of ARIA-H and ARIA-E (EMERGE and ENGAGE) [105,126].

	Mild	Moderate	Severe
**ARIA-H MB**	≤4 new MBs	5–9 new MBs	>9 new MBs
**ARIA-H SS**	1 focus	2 foci	>2 foci
**ARIA-E**	1 area < 5 cm	area 5–10 cm or	area > 10 cm
		≥2 locations each <10 cm	≥1 locations

Abbreviations: ARIA-E: amyloid-related imaging abnormalities–edema, effusion. ARIA-H: amyloid-related imaging abnormalities–hemorrhage; EMERGE: 221AD30 Phase 3 Study of Aducanumab (BIIB037) in Early Alzheimer’s Disease (EMERGE). NCT02484547; ENGAGE: 221AD301 Phase 3 Study of Aducanumab (BIIB037) in Early Alzheimer’s Disease (ENGAGE). NCT02477800; MB: microbleed; SS: superficial siderosis.

**Table 6:** Graduation of ARIA-E (SSAE 3 and 5) [105,126].

Size of ARIA-E	Number	SSAE 3	SSAE 5
No ARIA	0	0	0
<5 cm	monofocal	1 (mild)	1 (mild)
	multifocal	2 (moderate)	2 (mild +)
5–10 cm	monofocal	-	3 (moderate)
	multifokal	-	4 (moderate +)
>10 cm	monofocal	3 (severe)	5 (severe)
	multifocal		

Abbreviations: ARIA-E: amyloid-related imaging abnormalities–edema, effusion; SSAE: severity scale of ARIA-E.
